# The Gut–Bone Axis: A Systematic Review on the Potential Intervention Pathways for Bone Health

**DOI:** 10.3390/life16060909

**Published:** 2026-05-28

**Authors:** Tomás Cantón-Cordeiro, Saeka Shimochi, Miho Nakamura, Pere Puigbò

**Affiliations:** 1 Medicity Research Laboratory, Faculty of Medicine, University of Turku, 20520 Turku, Finland; 2 Department of Engineering, La Salle Campus Barcelona, University Ramon Llull, 08022 Barcelona, Catalonia, Spain; 3 Laboratory for Biomaterials and Bioengineering, Institute of Science Tokyo, Chiyoda, Tokyo 101-0062, Japan; 4 Department of Animal and Food Science, Faculty of Veterinary Medicine, Autonomous University of Barcelona, 08193 Bellaterra, Catalonia, Spain; 5 Department of Biology, University of Turku, 20014 Turku, Finland

**Keywords:** gut–bone axis, gut–brain–bone axis, bone health, osteoporosis, probiotics, gut microbiota, extracellular matrix, metabolites

## Abstract

Osteoporosis arises from disrupted bone remodeling, and growing evidence shows that gut microbiota and their metabolites have a major influence on skeletal health through the gut-bone and gut–brain–bone axes. In this systematic review, we synthesize findings from 932 studies to identify key microbial taxa, metabolites, and signaling pathways that modulate osteoblast and osteoclast activities. Short-chain fatty acids (SCFA), tryptophan-derived metabolites, and β-D-glucuronidase-related estrogen regulation emerge as central microbial mechanisms affecting bone formation and resorption. By integrating these data with the Phylobone extracellular matrix proteins database, we highlight Osteopontin and Cathepsin K as important downstream mediators linking microbial signals to bone matrix turnover. Probiotic strains (particularly *Lactobacillus rhamnosus* GG and *L. reuteri*) show potential to improve bone health through metabolic, immune, and endocrine pathways. Together, these findings outline a mechanistic framework connecting gut function to skeletal biology and identify promising microbiome-based targets for osteoporosis interventions.

## 1. Introduction

### 1.1. Background

The gut microbiome comprises trillions of microbial cells with a collective metabolic capacity far exceeding that of the human genome, leading many authors to consider it a functional microbial organ [1]. Through its extensive biochemical, immunological, and neuroendocrine interactions, the gut microbiome influences the physiology of multiple distal organs, including bone, brain, liver, and adipose tissue [2]. These cross-system interactions are structured through a series of bidirectional communication pathways collectively referred to as gut–organ axes. Within this framework, the gut–bone and gut–brain–bone axes have emerged as particularly relevant to skeletal homeostasis [3]. They integrate microbial metabolites, immune mediators, and neural signals that regulate osteoblast and osteoclast activities, thereby contributing to bone formation, bone resorption, and overall skeletal maintenance [4].

Given this extensive regulatory capacity, the gut–organ axes represent a promising avenue for both mechanistic insight and therapeutic intervention aimed at restoring homeostasis and preventing diseases such as osteoporosis. This review is timely because advances such as the Phylobone dataset (https://phylobone.com; accessed on 27 February 2026) now expand our understanding of how key bone extracellular matrix (ECM) proteins regulate bone remodeling [5,6]. These proteins retain clear signatures of being shaped by evolutionary locomotion transitions in vertebrates [6], which provide the basis for both their architectural roles in matrix organization and their regulatory influence on bone remodeling. Functional studies, including the differential expression rates of bone ECM proteins during mechanical-loading experiments [6], and the impact of signaling pathways that regulate the balance between bone formation and resorption to maintain skeletal homeostasis [7], further demonstrate how these protein families may contribute to the dynamic regulation of bone remodeling. Overall, these recent results enable the identification of a potential mechanistic framework that links microbial and neuroendocrine signals from the gut–bone and gut–brain–bone axes to ECM-mediated pathways with therapeutic potential.

### 1.2. Osteoporosis Disease

Osteoporosis is a metabolic bone disease characterized by decreased bone mass and deterioration of bone microarchitecture, leading to increased bone fragility and a higher risk of fractures [8]. The World Health Organization (WHO) defines it as a progressive systemic skeletal disease marked by low bone mass and structural deterioration of bone tissue. This condition predominantly affects individuals over the age of 60, with an estimated 200 million people affected globally [9]. Osteoporotic fractures are a major health concern, significantly impacting quality of life and contributing to morbidity and mortality, particularly due to hip, spine, and wrist fractures [10].

The primary cause of osteoporosis is an imbalance between bone resorption and bone formation, where bone resorption exceeds bone formation, leading to weakened bones. Osteoporotic fractures occur frequently, with an estimated 8.9 million fractures annually. The risk of fracture increases as bone mineral density (BMD) decreases [10]. The bone extracellular matrix (ECM) contains minerals deposited on highly crosslinked collagen fibrils and hundreds of non-collagenous proteins. Some of these proteins are key to the regulation of bone formation and regeneration via signaling pathways, playing important regulatory and structural roles. The process of osteogenesis, involving the differentiation of mesenchymal stem cells into osteoblasts that produce the bone ECM and regulate mineralization, is vital for maintaining bone health and strength [5].

Given the rising global life expectancy, the prevalence and impact of osteoporosis are expected to increase, underscoring the need for more effective and safer interventions [8]. Traditional therapies have largely focused on modifying bone remodeling directly, yet emerging evidence highlights the gut–bone and gut–brain–bone axes as additional biological routes that may be leveraged therapeutically [10]. These axes integrate microbial metabolites, immune mediators, and neuroendocrine signals that influence osteoblast and osteoclast activities, opening opportunities to modulate bone remodeling indirectly through the intestinal microbiome [11]. Probiotics, prebiotics, dietary components, and other microbiota-targeting strategies are therefore being explored as complementary approaches capable of influencing bone formation, mineralization, and inflammation [12,13]. By acting upstream of bone tissue, these gut-mediated pathways may provide novel avenues for improving osteoporosis prevention and treatment, particularly in populations with age-related dysbiosis or metabolic decline [14].

### 1.3. The Gut–Bone and Gut–Brain–Bone Axes

The gut–bone axis influences bone health through various pathways that are critical for bone homeostasis, including osteogenesis, osteoclastogenesis, and mineralization [15]. Osteogenesis involves the differentiation of mesenchymal stem cells into osteoblasts, which are responsible for bone formation [5]. Conversely, osteoclastogenesis refers to the differentiation of monocytes into osteoclasts, which are essential for bone resorption [5]. This dynamic process is crucial for bone remodeling and the maintenance of bone homeostasis. Both osteogenesis and osteoclastogenesis are regulated by several key proteins, including TSP1, TSP2, OPN, and MGP, which play vital roles in these processes [5,16].

The gut–brain–bone axis introduces an additional level of regulation by integrating neural, hormonal, and metabolic communication between the gastrointestinal tract, central nervous system, and skeletal tissues [14,17]. Signals from the gastrointestinal tract—transmitted through vagal pathways, gut-derived neurotransmitters, and circulating hormones—reach the central nervous system and subsequently influence bone turnover [17]. These signals can alter osteoblast and osteoclast behavior, illustrating how changes in gut physiology, diet, or microbiota composition can indirectly modify skeletal homeostasis through brain-mediated pathways [16,18].

Calcium and vitamin D supplementation, together with exercise—which directly strengthens bone through enhanced mineralization and mechanical loading—represent core strategies for improving skeletal health [5,6,19]. Beyond these direct effects, additional interventions such as probiotics, targeted dietary patterns, and specific pharmacological agents can further support bone metabolism by modulating the gut microbiota and influencing the gut–bone and gut–brain–bone axes [11,15]. In contrast, factors like the use of broad-spectrum antibiotics [20], poor-quality diets [21], and environmental contaminants (such as the use of herbicides [22]) may disrupt microbial homeostasis, leading to dysbiosis-mediated impairments in nutrient absorption, immune regulation, and neuroendocrine signaling that ultimately compromise bone health [23,24].

**Figure 1:**
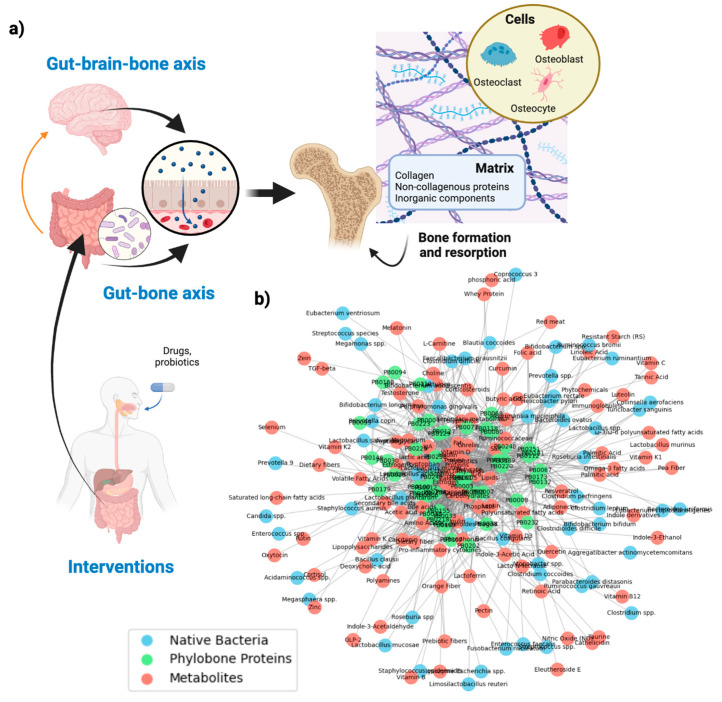
Illustration of the putative relationships between gut microbiota (blue nodes), metabolites (red nodes), and ECM proteins (green nodes). (**a**) The gut–bone and gut–brain–bone axes and potential intervention mechanisms by means of drugs and probiotics (Created in BioRender. Nakamura, M. (2026) https://biorender.com/7wf5wgh (accessed on 27 February 2026)). (**b**) Matrix of the relationship between gut microbial strains, metabolites and bone extracellular matrix proteins. A full size matrix is available in Figure S8.

Probiotics and pharmacological agents can modulate both the gut–bone and gut–brain–bone axes (Figure 1) [25]. By altering the production of microbial metabolites, probiotics can promote osteoblast activity, reduce osteoclastogenesis, and support a gut environment that favors bone maintenance [26]. This positions microbiome-targeted approaches as complementary strategies for improving bone health and potentially enhancing osteoporosis prevention and treatment [27].

## 2. Scope and Search Strategies in the Semi-Automated Systematic Review

**Figure 2:**
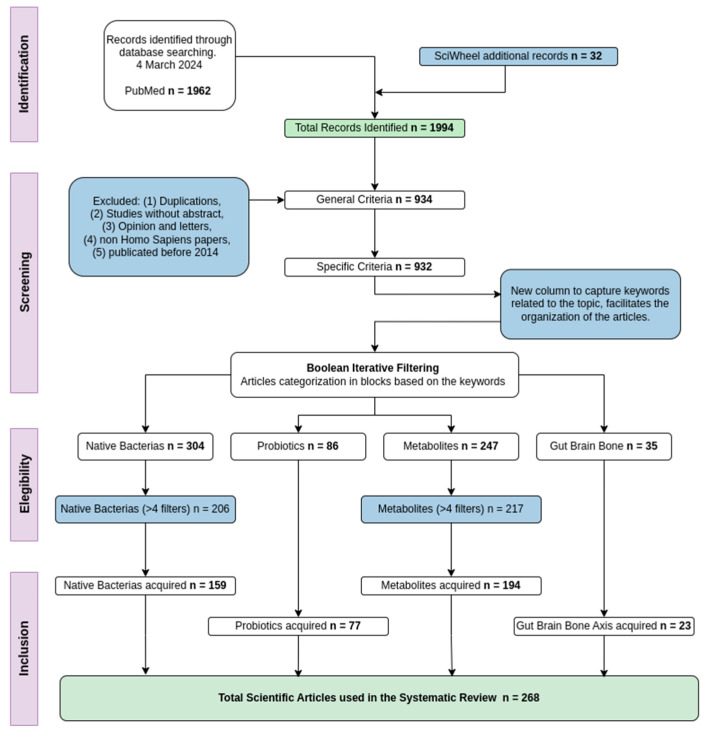
PRISMA flow diagram. This diagram visualizes the key steps in the systematic review, from the identification of articles to final inclusion of 268 relevant studies. Records identified in Pubmed with word search filters to construct the Supplementary Datasets S1–S4 (Box S1). First PRISMA workflow was implemented on 4 March 2024 for articles from 2014 to 2024. *Note*: A recent update of the workflow (25 April 2026), applied to 398 new articles published between 2024 and 2026 (Supplementary Datasets), and 118 passed all systematic workflow filters. None of the new articles that passed all filters show any additional metabolite, gut microbial strain or taxonomic group, nor probiotic strain or taxonomic group involved in the gut–bone or gut–brain–bone axes.

A systematic review was conducted to identify, evaluate, and synthesize current evidence on how gut microorganisms and their metabolites influence bone health through the gut–bone and gut–brain–bone axes. The review followed the PRISMA (Preferred Reporting Items for Systematic Reviews and Meta-Analyses) guidelines to ensure methodological transparency, reproducibility, and comprehensive reporting [28]. All stages of the process—including study identification, screening, eligibility assessment, and final inclusion—were performed according to PRISMA recommendations (Figure 2). This structured approach allowed a rigorous mapping of microbial taxa, metabolites, signaling pathways, and bone-related outcomes reported across the literature.

By synthesizing recent evidence, we map the mechanistic routes by which microbial products—such as short-chain fatty acids (SCFA), indole derivatives, and β-D-glucuronidase-related metabolites—modulate osteoblast and osteoclast activity via immune, endocrine, and neuroendocrine signaling. The review also integrates these mechanistic insights with bone extracellular matrix data from the Phylobone database to identify ECM proteins, such as Osteopontin and Cathepsin K, that act as downstream mediators of microbial and metabolic signals relevant to osteoporosis and bone health. The bidirectional interaction between the gut and bones is mediated by various metabolic, hormonal, and immunological factors. Overall, the review highlights how metabolic, hormonal, and immunological interactions between the gut and the skeletal system contribute to bone regeneration, maintenance of bone health, and the prevention of osteoporosis [5].

### 2.1. Automating a Systematic Review

Here, we provide a semi-automated systematic review—a rigorous and transparent method used to identify, evaluate, and synthesize studies—was conducted to explore the relations between the gut–bone axis and how intestinal bacteria and metabolites influence bone health [29].

The Preferred Reporting Items for Systematic Reviews and Meta-Analyses (PRISMA) method [28], were applied to ensure the systematic review is comprehensive and transparent. PRISMA is a set of guidelines designed to help researchers report systematic reviews and meta-analyses clearly and transparently, improving the quality of these studies (Figure 2). The steps were used in the PRISMA method: (1) Formulate the review question, (2) Define inclusion & exclusion criteria, (3) Develop a search strategy, (4) Select studies, (5) Extract data, (6) Assess study quality, (7) Analyze and interpret results, and (8) Disseminate findings.

### 2.2. Databases and Search Strategies

The search and selection of information for this systematic review were conducted through PubMed (https://pubmed.ncbi.nlm.nih.gov; accessed on 27 February 2026), a comprehensive database of scientific abstracts and medical citations, managed by the National Center for Biotechnology Information (NCBI) within the National Library of Medicine and the National Institutes of Health (NIH). PubMed provides access to a wide range of life sciences databases, containing over 3 billion records, which facilitates an exhaustive search of relevant scientific articles for any research topic 3.

Each article selected from PubMed undergoes a peer-review process (preprints were not included in our search), which is a standard practice ensuring the quality and relevance of the studies included in the database. This procedure adds an additional level of reliability to the articles chosen for the systematic review, guaranteeing a quality selection for the study topic 3.

The search strategy designed for this study was based on Boolean search terms to capture as much relevant literature as possible regarding the gut–bone axis. The search terms used were defined in Query 1.
(microbiota [Title/Abstract] OR microbiome [Title/Abstract]) AND (Bone [Title/Abstract])**Query 1.** Search performed in PubMed to identify articles.

This query was chosen with the aim of identifying studies discussing the relationship between the microbiota and bone health in humans. The search strategy was developed in consensus with the Phylobone research group, ensuring that it accurately captured the scope of relevant literature for this systematic review. The search initially yielded a total of 1962 articles on 4 March 2024. An updated search was performed on 26 February 2026 to capture recent discoveries on microorganisms and metabolites involved in the gut–bone and gut–brain–bone axes.

For the processing and management of the collected information, Sciwheel, a bibliographic management platform, was used. Sciwheel facilitated the review and suggestion of additional studies, resulting in 32 articles suggested by the tool, which were added to the dataset, obtaining a total of 1994 articles. With the support of Sciwheel, all articles were downloaded in BIB format, which facilitated the organization and portability throughout the systematic review.

### 2.3. Generation of Keywords

To ensure the relevance and quality of the information included in the systematic review, general inclusion and exclusion criteria were established. These criteria were applied to filter the initial database obtained from the PubMed search.

The following studies were excluded: (1) articles that did not have an abstract, since it is essential for the preliminary evaluation and for the generation of keywords that will be used later; (2) studies such as opinion articles or letters, as they do not provide relevant data for the research; (3) non Homo Sapiens studies, as the purpose of this review is to directly evaluate the interaction between human microbiome and bone health; (4) articles must contain the word ’gut’ to relate all the articles in the context of the gut–bone axis; (5) initially the articles were limited to studies published in the last ten years, from 2013 to 2024. A recent update of the dataset extended this search to 2026.

The application of these general inclusion and exclusion criteria allowed the optimization of the selection, facilitating the focus of efforts on the most relevant studies for the research objectives. As a result of applying these general criteria, the total number of articles was reduced to 932. This set of articles advanced to the next phase of the review, where specific inclusion and exclusion criteria were applied to build the data tables proposed in this research.

### 2.4. Specific Criteria

To ensure the relevance of the selected articles, a list of keywords related to bone health and the gut was established. This list includes microorganisms and probiotics, biological processes such as osteogenesis and osteoclastogenesis, also components such as proteins, peptides or metabolic pathways were included. The query was designed to fulfill these terms, allowing the filtering of articles that contain information directly relevant to the research objective. The keywords presented here serve as an illustrative set; the full list used in the study is more extensive and detailed.

Once the query was defined, a Python (version 3.14) algorithm was developed to compare the keywords column generated for each article with the query’s list of keywords. This algorithm acted as a filter, identifying articles that contained relevant terms. The final result was the creation of a new column for each article called Queried, which contains the keywords of interest present in each study.

### 2.5. Iterative Filtering for Classification

To achieve a more precise selection of the studies that will contribute to the construction of the four proposed tables, an iterative filter was implemented. This Boolean filter was executed through a Python script. The code facilitated the creation of detailed queries with keywords related to the specific theme of each table, using Boolean operators as ‘AND’ to restrict and ‘OR’ to include or classify the articles according to the keywords.

The ‘AND’ operators were used as restrictive filters, meaning that the articles must contain the keywords to be included. On the other side, the ‘OR’ operators were used as classificatory filters, if the articles contain the words, they will be added to the corresponding column, if they do not contain the ‘OR’ keywords, they will still be included as long as they meet with the ‘AND’ criteria.

Filters were used to capture the maximum number of relevant terms, ensuring that each table contains specific and pertinent studies for the research objectives (Box S1). These examples demonstrate the initial filtering process, but additional ‘OR’ filters were subsequently applied to comprehensively obtain the maximum number of articles belonging to each table. This iterative filtering approach ensures a thorough and targeted selection of articles for each category (Table S1).

### 2.6. Data Extraction

To analyze the studies and compile the relevant data for each of the four proposed tables, an iterative approach was used to prioritize the articles based on the number of keywords matched. The articles that contained the highest number of specific keywords were given higher priority and positioned at the top of the list for further analysis. This score ensured that the most relevant studies were reviewed first, maximizing the quality and relevance of the extracted data.

Efforts were made to obtain the full-text PDFs of the best articles. Access issues, such as institutional restrictions or private publications, were documented, and the corresponding articles were excluded from further analysis.

Once the relevant articles were obtained, an AI was employed to automate the extraction of the information. Each article was processed by attaching it to a specific prompt for each category. Native bacteria related to the gut–bone axis, probiotic strains, metabolites and metabolic pathways, or gut–brain–bone interactions. The AI system scanned the entire text of the article, extracting all relevant information in a narrative way.

After the AI summarized the information, the extracted data were organized into columns corresponding to each article. Depending on the category, these columns could include details or other relevant points. This information was then systematically added to a growing database, ensuring that all details were captured in a structured format.

The automation of this process allowed for the efficient and accurate compilation of a database that captured the complexity of the gut–bone axis interactions.

### 2.7. Quality Control

To ensure the accuracy and reliability of the results obtained in this study, a quality control was conducted. This process included the classification of articles depending on the number of keywords used in the iterative filtering that each article contained. Articles with the highest number of keywords were considered the most aligned with the criteria of the systematic review. To verify the accuracy of the results, the top 5 highest-scoring articles in each category were manually selected and analyzed, reaching a total of 20 articles reviewed.

For articles with fewer than 10 pages, the data extraction was nearly 100%, indicating an excellent performance in short documents. However, for articles with more than 15 pages, a loss of results was observed, especially towards data extracted located in the final pages. The manual review also revealed that the AI is more efficient at extracting data from tables than from figures or images. The AI processes text in tables better; extracting text from images presents greater challenges due to different format and quality (Figure S1).

The categories of bacteria and probiotics showed a higher accuracy rate compared to the category of metabolites. A possible explanation is that the scientific names of bacteria are often italicized, which might make it easier for the AI to identify text with special formatting.

The overall accuracy rate is around 80%, indicating high precision and validating the method used for data extraction in this systematic review (Figure S2).

## 3. Results and Discussion

### 3.1. Potential Targets Within the Gut–Bone Axis

The gut microbiota releases metabolites that travel through the bloodstream and have a physiological impact on most organs of the organism [2,30]. A healthy gut microbiota is essential for maintaining systemic homeostasis and strongly influences bone health by regulating bone formation and resorption through microbial metabolites, immune modulation, and hormone-dependent signaling pathways [2]. However, these interactions can be helpful or harmful, depending on the microbes and metabolites involved. The gut–bone axis highlights the importance of having a microbial balance for maintaining skeletal health, with some native bacteria and their metabolites promoting osteogenesis, while others driving to osteoclastogenesis and bone resorption [31,32,33]. The following results explore how native gut microbes and their metabolites may impact or regulate these processes in the bone (Figure S3).

#### 3.1.1. Native Bacteria Supporting Bone Formation

Several gut bacteria play a significant role in promoting osteogenesis, primarily through the production of short-chain fatty acids (SCFAs) and modulation of the immune system (Figure S4 and Supplementary Dataset S1) [34,35,36]. E.g., *Lactobacillus acidophilus* promotes bone formation by balancing the Treg-Th17 cell axis, which reduces inflammation and supports osteoblast activity, crucial for bone health [37]. Similarly, *Bifidobacterium longum* enhances BMD by increasing the bioavailability of polyphenols and producing butyrate, a potent anti-inflammatory SCFA that supports bone health [38]. The *Lachnospiraceae* family, including *Eubacterium* and *Roseburia*, also contribute significantly to bone remodeling through butyrate production, which enhances bone formation and reduces bone resorption. Additionally, *Faecalibacterium prausnitzii* is a major butyrate producer, known for its anti-inflammatory properties, which positively impact bone health by promoting bone formation [39]. Actinobacteria, particularly those within the genus *Bifidobacterium*, support bone health through SCFA production, especially butyrate, which modulates immune responses and promotes bone formation [40].

#### 3.1.2. Native Bacteria Promoting Bone Resorption

In contrast, certain gut bacteria are associated with promoting osteoclastogenesis and bone resorption, often through inflammatory pathways or the production of harmful metabolites (Figure S4 and Supplementary Dataset S1) [41,42]. *Escherichia coli*, particularly pathogenic strains, can produce TMA from dietary nutrients [43], which the liver converts into trimethylamine-N-oxide (TMAO). Elevated TMAO levels have been shown in experimental models to inhibit osteoblast differentiation and promote osteoclast-mediated bone resorption, suggesting a potential role in bone loss [4,41,42]. Additionally, *Bacteroides fragilis* and other species within the genus are more abundant in osteoporosis cohorts, suggesting a role in bone metabolism through inflammatory or metabolite pathways, although some strains like *Bacteroides ovatus* have been linked to bone strength in different contexts [44]. *Prevotella copri* is particularly noted for its association with increased severity of rheumatoid arthritis, a condition that leads to significant bone erosion and highlights the potential for *Prevotella* spp. to negatively impact bone health through systemic inflammation [45]. *Clostridium difficile* is another bacterium linked to gut dysbiosis and systemic inflammation [46], both of which can negatively affect bone health, although *Clostridium butyricum* may support osteogenesis under certain conditions [47]. Although Firmicutes are generally associated with maintaining gut health [48,49], certain species within this phylum can negatively influence bone health under inflammatory conditions [50], highlighting their context-dependent and dual roles [51].

#### 3.1.3. Metabolites Involved in Bone Health

The most relevant metabolites are grouped and described according to their categories, their primary metabolic pathways, and their documented effects on bone health (Figure S5 and Supplementary Dataset S2).

##### SCFAs

SCFAs—including butyrate, propionate, and acetate—are produced by the fermentation of complex carbohydrates by the gut microbiota [52]. These SCFAs activate the Wnt/β-catenin pathway, promoting the differentiation of mesenchymal stem cells into osteoblasts, thereby enhancing bone formation [53]. Additionally, butyrate reduces systemic inflammation by inhibiting NF-*κ*B signaling, protecting against bone loss [54].

##### Vitamins and Minerals

Various micronutrients regulated by the gut microbiota, or influenced by overall gut health, play a significant role in bone health [55]. Vitamin D is fundamental for calcium absorption and proper bone mineralization, with its activation and metabolism closely linked to intestinal function [56]. Adequate vitamin D levels are therefore crucial to maintain optimal calcium absorption and bone density. Other micronutrients, such as selenium and vitamin C [57], contribute to bone health primarily through their antioxidant effects and their influence on collagen synthesis, which is vital for maintaining bone matrix structure and strength.

##### Amino Acid-Derived Metabolites

Some metabolites derived from the metabolism of amino acids, such as tryptophan and phenylalanine, have a notable impact on bone health [58]. Picolinic acid (PIC), a product of tryptophan catabolism, has anabolic effects that promote bone formation [59]. Similarly, indoles, also derived from tryptophan, modulate the immune system and possess anti-inflammatory properties, which help protect against bone loss by reducing systemic inflammation [60].

##### Hormones and Growth Factors

The interaction between the gut and the regulation of hormones and growth factors significantly influences bone health [61]. Melatonin known for its antioxidant and anti-inflammatory effects, positively impacts bone formation by activating pathways such as Wnt/β-catenin signaling, which are essential for osteoblast differentiation and function [62]. Insulin-like growth factor 1 (IGF-1) serves as a critical connection between gut health and bone density, influencing bone growth and remodeling through circulating IFF-1 levels [40].

##### Inflammatory and Toxic Metabolites

Some metabolites can negatively affect bone health, particularly those that induce inflammation [63]. Lipopolysaccharides (LPS), which act as bacterial endotoxins, contribute to bone loss by stimulating proinflammatory cytokine production and osteoclastogenesis [64,65]. TMAO, produced by the gut microbiota from L-carnitine, is associated with increased bone loss, especially in weight loss contexts, due to its pro-inflammatory effects [66].

#### 3.1.4. Probiotics as Potential Intervention Treatment for Osteoporosis and Bone Related Diseases

A wide range of bacteria species have been studied on the basis of their probiotic potential in the context of osteoporosis and bone-related diseases (Table 1 and Figure S6). Notably, *Lactobacillus reuteri* is the most cited species (24 mentions), followed by *L. rhamnosus* and *L. casei*, all of which have been associated with improved bone health via enhanced calcium absorption [67]. Other species, including *L. acidophilus* and *L. plantarum*, also appear and have been associated with enhanced osteogenesis and preserved bone homeostasis [68]. Among the most frequently mentioned probiotic strains in the included studies, *L. rhamnosus* GG (LGG) stands out as the most cited. It is known for improving gut health, preventing TNF-α mucosal damage, and enhancing calcium absorption. *Lactobacillus reuteri ATCC 6475* is notable for its ability to improve BMD in postmenopausal women [69]. This strain suppresses the expression of pro-inflammatory and pro-osteoclastogenic cytokines, reducing bone resorption and contributing to immune system modulation. *Bacillus subtilis C-3102* stands out for its ability to increase BMD, inhibit bone resorption, and improve calcium absorption [70]. This probiotic supports osteoblast proliferation and differentiation through butyrate production. *Lactobacillus casei Shirota* participates in the production of low molecular weight metabolites that contribute to anti-inflammatory effects, which is crucial for fracture healing and reducing markers of osteoarthritis [45].

Overall, the results show the importance of *Lactobacillus* and *Bifidobacterium* species in promoting bone health through different metabolic pathways and the production of specific metabolites (Figure S6, Table 1, and Supplementary Dataset S3).

#### 3.1.5. Case of Study: *Lactobacillus rhamnosus* GG (LGG)

LGG influences several metabolic pathways essential for maintaining bone density and preventing bone-related diseases, including SCFA production, Wnt/β-catenin signaling, and immune modulation (Table 2). Furthermore, LGG’s enhances vitamin D absorption [75] and reduces inflammation through the inhibition of NF-κB highlighting its potential as a probiotic therapy [38,48,68]. Specifically, LGG has been identified to participate in the pentose and glucuronate interconversion metabolic pathway (Supplementary Figure S7). Within this pathway, the enzyme beta-D-glucuronidase plays a crucial role in the metabolism of glucuronides. LGG modulates the activity of this enzyme, affecting the availability of glucuronides, which are important for the excretion of metabolites and the regulation of estrogen levels in the body [73]. Overall, these observations position LGG-mediated glucuronide metabolism as a meaningful pathway through which gut microbes may support estrogen-related bone health.

### 3.2. Potential Targets Within the Gut–Brain–Bone Axis

The gut–brain–bone axis represents a complex network of communication between the gut, the central nervous system, and the skeletal system [17]. This axis integrates biochemical and microbial signals from the gut that can influence brain function and bone health (Table 3). Various metabolic pathways and their metabolites, along with the bacteria that produce or modify them, have been mapped within this axis. Metabolites such as SCFAs, neurotransmitters like GABA, and bone-derived hormones like osteocalcin may play critical roles in modulating inflammatory processes, immune responses, and bone homeostasis (Table 3).

A central pathway in bone resorption is the RANKL–RANK signaling cascade, which drives osteoclastogenesis [81]. The inhibition of this pathway by butyrate, not only reduces bone loss but also promotes bone formation [82]. This dual action makes it a particularly promising target for therapeutic interventions. Serotonin signaling is another area to explore, as this neurotransmitter influences osteoblast and osteoclast activities, though its exact role in bone remodeling remains unclear [77]. The cross-talk between serotonin production in the gut and its effects on the skeletal system could open new avenues for research, particularly in understanding how stress and mental health conditions contribute to bone diseases [77]. Because only 23 studies addressed the gut–brain–bone axis, the available evidence remains limited, and further research will be needed to clarify its mechanisms and relevance.

### 3.3. Bone ECM Proteins as Mediators of the Gut–(Brain)–Bone Axes

The bone ECM consists of ~40% organic and ~60% inorganic components, with the organic fraction largely composed of collagen and a broad diversity of non-collagenous proteins that regulate mineral deposition, osteoblast and osteoclast activities, and overall bone remodeling [5]. While collagen provides structural support, non-collagenous ECM proteins act as key modulators of signaling pathways central to skeletal homeostasis [83,84]. In the context of the gut–bone and gut–brain–bone axes, our hypothesis is that non-collagenous bone ECM proteins serve as downstream mediators of gut-derived microbial, immune, and neuroendocrine signals. This framework places non-collagenous ECM proteins as the likely end-effectors through which gut-derived signals are converted into molecular responses within bone tissue.

Osteopontin stands out as the most extensively investigated non-collagenous ECM proteins, making them compelling candidates for downstream mediators through which gut-derived signals may influence bone remodeling (Table 4 and Supplementary Table S2) [85]. Cathepsin K is a critical osteoclast-derived protease responsible for degradation of bone matrix and represents a central effector of bone resorption [86]. The dominance of cathepsin K and osteopontin suggests that these proteins play important roles in bone remodeling and resorption processes [87]. Other proteins such as fibroblast growth factor 23 act as endocrine regulators of mineral metabolism, while matrix Gla protein contributes to the regulation of ECM mineralization [88], pointing to emerging areas of interest for future investigation (Supplementary Table S2). Nevertheless, the Phylobone database provides a comprehensive catalog of 255 bone ECM proteins in humans, offering a valuable framework for comparative and functional analyses [5]. Recent studies also suggest that Fetuin-A may play a key role in mediating mechanical loading in bone [6], pointing to additional ECM components with potential regulatory relevance. Further studies should clarify whether—and how—such ECM proteins integrate signals from the gut microbiota and participate in the gut–bone and gut–brain–bone axes.

#### 3.3.1. Osteopontin

Osteopontin is a key non-collagenous ECM protein involved in bone remodeling and immune regulation. Its main action in the body is to mediate the adhesion of osteoclasts to the bone mineralized matrix, facilitating bone resorption and remodeling [48]. Additionally, beyond its structural role, osteopontin participates in inflammatory pathways such as NF-*κ*B and MAPK cascades, and interacts with the RANK/RANKL system to modulate osteoclast activity [89,90]. This protein is influenced by several substrates, including butyric acid and gangliosides present in milk. Some probiotics involved in its activity are *Faecalibacterium prausnitzii*, *Lactobacillus reuteri*, *Bifidobacteria*, and *Dialister* [48]. Given its central role in osteoclast function and immune-bone interactions, osteopontin represents a potential downstream mediator linking gut-derived inflammatory signals to bone remodeling [90]. However, its effects are context-dependent and may contribute to either physiological remodeling or pathological bone loss depending on the inflammatory conditions.

#### 3.3.2. Cathepsin K

Cathepsin K is a lysosomal cysteine protease that participates in the degradation of type I collagen during bone resorption (Table 4). Its expression increases during osteoclast differentiation, primarily under the control of RANKL signaling. Conditions associated with chronic low-grade inflammation such as obesity are characterized by elevated pro-inflammatory cytokines such as TNF-*α* and IL-1*β*, which upregulates Cathepsin K expression, resulting in a reduction in BMD [88]. Additionally, microbe-associated molecular patterns (MAMPs) also modulate Cathepsin K activity indirectly. LPS from Gram-negative bacteria activates the TLR4 receptor, increasing the production of inflammatory cytokines like IL-1*β* and TNF-*α*, which in turn stimulates osteoclast activity and bone resorption. On the other hand, SCFAs, products of bacterial metabolism in the gut, can inhibit osteoclast differentiation at low doses, promoting osteogenic activity and suggesting a potential protective effect on bone health [91].

**Table 4:** Metabolic pathways related to Osteopontin Cathepsin K and their impact on bone health.

Metabolic Pathway	Effect in Bone Health and Metabolites Involved	Related Microbiota	References
**Osteopontin**			
**NF-** κ **B Signaling**	Promotes osteoclastogenesis by activating osteoclasts, leading to bone resorption. Inflammatory cytokines like TNF-α enhance the expression of osteopontin, further promoting resorption.	*Lactobacillus reuteri*, *Bifidobacteria*	[92]
**RANK/RANKL Signaling**	Facilitates osteoclast differentiation and activation, leading to bone resorption. Osteopontin binds to integrin receptors on osteoclasts, enhancing their adhesion to bone matrix.	*Faecalibacterium prausnitzii*, *Lactobacillus reuteri*	[48,92]
**MAPK Pathway**	Regulates osteoclast activity and expression of osteopontin, contributing to bone resorption. It also impacts osteoblast activity through the production of growth factors like TGF-β.	*Faecalibacterium prausnitzii*, *Dialister*	[48]
**TGF-** β **/BMP Pathway**	Modulates osteoblast differentiation and activity, promoting bone formation. Osteopontin acts as an anti-apoptotic factor for osteoblasts, favoring continuous bone formation.	*Faecalibacterium prausnitzii*, *Bifidobacteria*	[93]
**WNT Signaling**	Enhances osteoblast differentiation and bone formation through the regulation of gene expression related to bone development. Osteopontin is involved in maintaining osteoblast survival.	*Lactobacillus reuteri*, *Bifidobacteria*	[48,93]
**TAZ/IHH Pathway**	Contributes to the regulation of osteogenesis and the maintenance of bone matrix, supporting the role of osteopontin in bone formation.	*Faecalibacterium prausnitzii*	[48]
**Cathepsin K**			
**Inflammation induced by obesity**	TNF-α, IL-1β: These cytokines increase Cathepsin K activity, enhancing collagen degradation and osteoclast differentiation, leading to increased bone resorption and decreased BMD.	Promotes osteoclastogenesis, leading to increased bone resorption and decreased BMD.	[88]
**Signaling by LPS**	IL-1β, IL-6, TNF-α: LPS induces these cytokines, which stimulate Cathepsin K activity, boosting osteoclast activity and accelerating bone matrix breakdown.	Promotes osteoclastogenesis, leading to increased bone resorption.	[91]
**MAMPs**	TRAP, MMP-9, Runx2, osterix: SCFAs, products of bacterial metabolism, inhibit osteoclast differentiation at low doses, reducing Cathepsin K activity, thus supporting bone formation and promoting osteogenic activity.	Promotes osteogenic activity, providing a protective effect on bone health.	[91]

## 4. Limitations

This semi-automated systematic review synthesizes the current human evidence supporting a connection between the gut microbiota and bone health and discusses potential connections between microbiota metabolites and regulation of the bone extracellular matrix. However, there are several limitations to note here. While the review only considers human data, the vast majority of these associations remain correlative and thus are subject to confounders such as nutrition, age, lifestyle, or systemic inflammation. Moreover, the use of observations from human subjects makes it hard to elucidate the underlying biological mechanisms that can explain these associations. The diversity in study design, population characteristics, and methods of microbiome evaluation is likely to contribute to inconsistencies within this area. Thus, there are currently no causative and mechanistic insights available in this area. Future studies need to utilize longitudinal cohorts and controlled experiments among humans to provide stronger support for causal inference. The implementation of multi-omics and standardization of outcome measurements may help to achieve greater reproducibility and translational potential.

## 5. Conclusions

This review brings together growing evidence that the gut is far more than a digestive organ—it is an upstream regulator of skeletal biology. Microbial metabolites, immune and neuroendocrine signals generated in the gut can influence how bone is formed, remodeled, and maintained throughout life. By examining these interactions across the gut–bone and gut–brain–bone axes, we highlight a biological landscape where intestinal health, brain signaling, and bone remodeling are deeply interconnected.

Placing these findings alongside the Phylobone dataset gives this field a new layer of clarity. Bone ECM proteins emerge as crucial intermediaries linking microbial activity to skeletal outcomes. They act not only as the scaffold of bone but as regulatory hubs that shape osteoblast and osteoclast behavior. This adds mechanistic depth to the idea that the microbiome may influence bone not indirectly, but through defined ECM-linked pathways (such as Osteopontin and Cathepsin K). However, further studies are needed to confirm these potential intervention pathways. In addition, further studies should reveal whether additional ECM proteins, such as Fetuin-A [6], could serve as mediator proteins in the gut–bone and gut–brain–bone axes, especially in the context of mechanical loading [6].

Overall, the microbiome represents a promising, still underused therapeutic frontier in osteoporosis and other bone-related disorders. Targeting gut bacteria, their metabolites, or the host pathways they modulate could complement existing treatments, support healthier aging, and open new possibilities for personalized interventions. At the same time, our work shows that modern computational tools (such as the semi-automated literature synthesis) can accelerate discovery in this rapidly evolving field, helping researchers navigate the expanding complexity of microbiome-bone interactions. Furthermore, this study points to gut homeostasis as a potential contributor to bone health, but its clinical relevance will need to be assessed through focused interventions and carefully designed studies.

## Figures and Tables

**Table 1:** Probiotic strains and their effects on bone health.

Probiotic Strain	Effect on Bone Health	Metabolic Pathways and Metabolites	References
*Lactobacillus rhamnosus* GG	Improves gut barrier health, prevents TNF-α induced mucosal damage, enhances calcium absorption.	Enhances mucin expression, produces SCFAs increasing calcium solubility and absorption. Protein HM0539 involved.	See Table 2
*Lactobacillus casei* Shirota	Enhances gut health, reduces inflammation, improves fracture healing, reduces osteoarthritis biomarkers.	Produces low molecular weight metabolites, increases IL-10, reduces TNF-α, IL-6, and IL-12, aiding in anti-inflammatory effects.	[45,70]
*Bacillus subtilis* C-3102	Increases BMD, inhibits bone resorption, improves calcium absorption.	Increases uNTx and TRACP-5b, enhances gut microbiota, increases butyrate production which supports osteoblast proliferation and differentiation.	[69,70,71]
*Lactobacillus reuteri* ATCCPTA 6475	Reduces BMD loss in older women.	Modulates gut microbiota, suppresses the expression of pro-inflammatory and pro-osteoclastogenic cytokines reducing bone resorption.	[69]
*Lactobacillus plantarum* GKM3 and DSM 15312	Inhibits bone loss, promotes osteogenesis, decreases osteoclastogenesis.	Produces SCFAs, regulates VDR and TRPV6 for calcium absorption, modulates claudin-2 for intestinal permeability.	[69]
*Lactobacillus paracasei* DSM 13434	Protects against rapid BMD loss in postmenopausal women.	Produces SCFAs, reduces intestinal permeability, decreases pro-inflammatory cytokines like TNF-α.	[69]
*Lactobacillus intestinalis* YT2	Alleviates menopausal symptoms, including bone density loss.	Restores gut microbiota composition, enhancing overall gut health and indirectly supporting bone health.	[71]
*Lactobacillus plantarum* NK3	Alleviates bacterial vaginosis and osteoporosis.	Suppresses NF-κB/TNF-α pathway, reducing inflammation and promoting bone health.	[72]
*Lactobacillus reuteri* NCIMB 30242	Increases serum vitamin D levels, crucial for bone health.	Increases circulating 25-hydroxyvitamin D, supporting bone mineralization and health.	[72]
*Bifidobacterium lactis* Probio-M8	Improves bone metabolism, increases vitamin D3, decreases PTH and procalcitonin, enhances calcium absorption.	Involves carbohydrate metabolism pathways, enhances gut microbial interactions, increases SCFA-producing bacteria and choline-phosphate cytidylyltransferase.	[73]
*Streptococcus salivarius* W24	Inhibits periodontopathogens, maintains immune homeostasis.	Targets IL-6 and IL-8 pathways, producing bacteriocins that suppress pathogen growth.	[71]
*Tenericutes* ML615J-28, 124-7	Reduction in abundance in Polycystic Ovary Syndrome (PCOS) patients, potentially improves bone health.	Specific metabolic pathways not clearly defined, but reduction in abundance correlates with improved bone health.	[71]
*Bifidobacterium lactis* HN019	Reduces IL-1β, RANKL-OPG ratio, TNF-α, and IL-6, disrupts Gram-negative bacteria membrane, reduces *P. gingivalis* adhesion.	Regulates the immune system through organic acids like lactic acid, contributing to anti-inflammatory effects.	[74]

**Table 2:** Summary of metabolic pathways, metabolites, and effects of *Lactobacillus rhamnosus* GG (LGG) on bone health.

Metabolic Pathways and Involved Metabolites	Effects on Bone Health	How LGG Influences	References
**Production of SCFAs (Butyrate, propionate, acetate)**	Promotes the differentiation of mesenchymal stem cells into osteoblasts, improves bone formation, and reduces bone resorption.	Increases the production of SCFAs, especially butyrate, which activates the Wnt/β-catenin pathway and reduces inflammation by inhibiting NF-κB.	[38,48,60,68,72,73]
**Wnt/** β **-catenin (Butyrate)**	Stimulates the accumulation of β-catenin, promoting the proliferation and differentiation of stem cells into osteoblasts.	Increases the production of butyrate, which activates the Wnt/β-catenin pathway to promote bone formation.	[38,73]
**NF-** κ **B (Butyrate)**	Reduces inflammation, creating a favorable environment for bone formation by inhibiting osteoclast activation.	Producing butyrate, inhibits NF-κB signaling, which decreases inflammation and protects bone mass.	[38,72,73]
β **-D-glucuronidase (Estrogens)**	Regulates estrogen levels, essential for maintaining bone density in postmenopausal women.	Reduces β-D-glucuronidase activity, limiting estrogen reabsorption and reducing the risk of reproductive cancers, maintaining a healthy estrogen balance.	[38,48,73]
**Tryptophan Metabolism (Indoles)**	Modulates the immune system, decreases inflammation, and supports bone health.	Metabolizes tryptophan to produce indoles, which have anti-inflammatory effects and contribute to beneficial immune modulation for bone health.	[60]
**Glycolysis (Lactate)**	Maintains a healthy intestinal environment that indirectly supports bone health.	Produces lactate through glycolysis, helping maintain an appropriate intestinal pH, favoring the presence of beneficial bacteria that support bone health.	[60]
**Vitamin D Absorption (Vitamin D)**	Improves calcium absorption, essential for bone mineralization and overall bone health.	Increases the expression of the vitamin D receptor in intestinal cells, improving calcium absorption.	[68]
**Immune Modulation (IL-10, TGF-** β **)**	Decreases osteoclast activity and promotes bone formation by increasing the production of anti-inflammatory cytokines.	Promotes the expansion of Treg cells that secrete IL-10 and TGF-β, modulating the immune response to protect against bone loss.	[38,60,72,73]
**Strengthening of the Intestinal Barrier (SCFAs, Butyrate)**	Strengthens the intestinal barrier, reduces intestinal permeability, and protects against systemic inflammation that negatively affects bone health.	Produces SCFAs like butyrate, which improves intestinal barrier function, reducing endotoxin translocation and systemic inflammation.	[38,60,72]
**Regulation of Osteoprotegerin (OPG)**	Inhibits osteoclast formation and reduces bone resorption, promoting bone formation.	Increases the expression of OPG, a decoy receptor that blocks the interaction of RANKL with RANK, thus decreasing osteoclastic activity.	[60]

**Table 3:** Metabolic pathways, effects, involved metabolites and participating microbiota in the gut–brain–bone axis.

Metabolic Pathway	Effect and Relation with the Gut–Brain–Bone Axis	Participating Microbiota	References
RANKL and TRAP5 Pathways	Inhibition of these pathways by butyrate reduces osteoclastogenesis, promotes bone formation, improves bone mineral density, and reduces bone loss.	*Lactobacillus reuteri*, *Lactobacillus plantarum*, *Lactobacillus paracasei*	[76]
Anti-Inflammatory Pathways	SCFAs (butyrate) reduce inflammatory cytokines (IL-6 and TNF-α), improving bone and brain health by reducing systemic inflammation.	*Lactobacillus reuteri*, *Bifidobacterium longum*, *Faecalibacterium prausnitzii*	[76,77]
Treg-Th17 Modulation	GABA and butyrate regulate the differentiation of Treg and Th17 cells, balancing the immune response, which is essential for maintaining bone and brain health.	*Lactobacillus rhamnosus* (JB-1)	[77,78]
IGF-1 Signaling Pathway	SCFAs enhance IGF-1 signaling, promoting bone formation and growth by connecting gut and brain signaling, which improves bone density and strength.	*Bifidobacterium longum*, *Bacteroides* spp.	[79]
Serotonin Signaling	Serotonin regulates osteoblast and osteoclast activities, influencing bone density and quality through central and peripheral signaling, ensuring balanced bone remodeling.	*Escherichia coli*	[77]
G-protein-coupled receptors (GPCR)—GPR41, GPR43, GPR109A	SCFAs interact with GPCRs to regulate renin release and blood pressure, indirectly benefiting bone health through anti-inflammatory effects.	*Lactobacillus murinus*, *Bacteroides* spp.	[79]
Olfactory Receptor 78 (Olfr78) Involved metabolite: SCFAs (Propionate)	Propionate promotes vasodilation by altering Olfr78 and GPR41 activity, contributing to an acute hypotensive response and indirectly benefiting bone health.	*Bacteroides* spp.	[80]
GABA Production	GABA regulates brain function, reduces anxiety, and improves mood, indirectly benefiting bone health by reducing stress-related bone loss.	*Lactobacillus rhamnosus* (JB-1), *Streptococcus thermophilus*	[78,79]
Osteocalcin Production	Osteocalcin, along with vitamin K and SCFAs, directly influences bone formation and homeostasis, preventing fractures and maintaining bone health.	*Lactobacillus reuteri*, *Bacteroides* spp., *Faecalibacterium prausnitzii*	[76,77,78]
Neuromodulator Production	Neurotransmitters like serotonin and dopamine regulate mood and behavior, impacting bone health by influencing the stress response and ensuring balanced bone remodeling.	*Escherichia coli*, *Lactobacillus* spp.	[78]
Dietary Fiber Fermentation	SCFAs produced during fiber fermentation regulate inflammation, improve intestinal permeability, and enhance calcium absorption, all critical for bone health.	*Faecalibacterium prausnitzii*, *Bacteroides* spp., *Lactobacillus* spp., *Clostridium* spp.	[77,79]
Corticotropin (CRH) Signaling	CRH plays a key role in regulating the stress response, with chronic stress negatively affecting both brain and bone health. SCFAs and other metabolites help mitigate these effects.	*Lactobacillus* spp., *Bifidobacterium* spp., *Firmicutes*, *Tenericutes*	[76,77]

## Data Availability

All data are available in the manuscript, Supplementary Materials and Supplementary Datasets.

## Supplementary Materials

The following supporting information can be downloaded at: https://www.mdpi.com/article/10.3390/life16060909/s1. Box S1. Search queries in Pubmed; Figure S1. Comparison of results obtained by AI and manual review; Figure S2. Total accuracy percentage in each category of tables; Figure S3. Illustration of the gut-bone axis and its interaction with the gut-brain axis in bone homeostasis; Figure S4. Top most frequent native bacteria in the human gut microbiota; Figure S5. Top most frequent metabolites; Figure S6. Most frequent probiotics (Genus and Species). This figure shows the most frequently identified probiotic species. Lactobacillus (blue), Bifidobacterium (green), Streptococcus (red), and Bacillus (purple); Figure S7. LGG metabolic pathways image. Pentose and glucuronate interconversions pathway (map00040); Figure S8. Phylobone network illustrates the relationships between gut microbiota (blue nodes), metabolites (red nodes), and ECM proteins (green nodes); Table S1. Summary of articles analyzed. For native bacteria and metabolic pathways, articles with more than 4 keyword matches were prioritized; Table S2. Frequency of relevant proteins according to the Phylobone database, with corresponding PBID; Dataset S1. Microbiot; Dataset S2. Metabolites; Dataset S3. Probiotics; Dataset S4. Bone ECM Proteins.
