# Supplementary material for: The Gut–Bone Axis: A Systematic Review on the Potential Intervention Pathways for Bone Health
Source: Life (Basel). 2026 May 28;16(6):909. doi: 10.3390/life16060909 (PMC13300829; doi:10.3390/life16060909)
Supplement: Supplementary file 1 [file life-16-00909-s001.zip › 2_Canton_cordeiro_Supplementary_Material-2.docx]

**SUPPLEMENTARY MATERIAL**

**The Gut-Bone Axis: Potential Intervention Pathways for Bone Health**

Tomás Cantón-cordeiro^1^, Saeka Shimochi^1^, Miho Nakamura^1,2,3,&*^ and ‪Pere Puigbò^4,5,&*^

^1^ Medicity Research Laboratory, Faculty of Medicine, University of Turku; Turku, Finland.

^2^ Department of Engineering, La Salle Campus Barcelona, University Ramon Llull, Barcelona, Catalonia, Spain.

^3^ Laboratory for Biomaterials and Bioengineering, Institute of Science Tokyo, Chiyoda, Tokyo, Japan..

^4^ Department of Animal and Food Science, Faculty of Veterinary Medicine, Autonomous University of Barcelona, Bellaterra, Catalonia, Spain

^5^ Department of Biology, University of Turku; Turku, Finland

^&^ These authors jointly supervised this work.

^*^ Corresponding authors:

Pere Puigbò. Email: [pere.puigbo@uab.cat](mailto:pere.puigbo@uab.cat)

Miho Nakamura. Email: [miho.nakamura@salle.urv.cat](mailto:miho.nakamura@salle.urv.cat)

## Supplementary boxes

**Box S1.** Search queries in Pubmed

| **Dataset 1: Native bacteria related to the gut-bone axis.** Search string for gut microbiota and bone health. This table focuses on identifying bacterias in the gut that are related to bone health. This process reduced the list to 304 articles.  \| ("Gut" AND "Bone") AND ("Bacteria" OR "Microbiota" OR "Strain" OR "Osteogenesis" OR "Osteoclastogenesis" OR "Lactobacillus" OR "Bifidobacterium" OR "Faecalibacterium" OR "Bacteroides" OR "Streptococcus" OR "Clostridium" OR "Eubacterium" OR "Akkermansia" OR "Propionibacterium" OR "Bone Health") \| **Query 1** \| \| --- \| --- \|  **Dataset 2: Probiotic strains and bone health.** Search string for probiotics and bone health. This table seeks information on external probiotic strains and their impact on bone health. This filtering resulted in 86 articles.  \| ("Probiotics" AND "Gut" AND "Bone") AND ("Strain" OR "Osteoblast" OR "Osteoclast" OR "Bacillus" OR "Lactobacillus" OR "Bifidobacterium" OR "Saccharomyces" OR "Probiotic" OR "Osteogenesis") \| **Query 2** \| \| --- \| --- \|  **Dataset 3: Metabolites and metabolic pathways.** Search string for metabolites and bone health. This table includes articles detailing how proteins and other molecules affect bone regeneration and destruction, reducing the total to 247 selected articles.  \| ("Gut" AND "Bone") AND ("Proteins" OR "Peptide" OR "Molecule" OR "Metabolite" OR "Cytokine" OR "Hormone" OR "Signaling" OR "Pathway" OR "Biotic" OR "Osteogenesis" OR "Bone Formation" OR "Bone Resorption" OR "Bone Health") \| **Query 3** \| \| --- \| --- \|  **Dataset 4: Gut-brain-bone interactions**. Search string for gut-brain-bone interactions. This table focuses on studies that connect the gut microbiota, brain, and skeleton. This filtering led to the identification of 35 relevant articles.  \| ("Gut" AND "Bone" AND "Brain") AND ("Protein" OR "Hormone" OR "Matrix" OR "Extracellular" OR "Health" OR "Bacteria" OR "Microbiota" OR "Neuro" OR "Path") \| **Query 4** \| \| --- \| --- \| |
| --- | --- | --- | --- | --- | --- | --- | --- | --- |

##

## Supplementary Figures


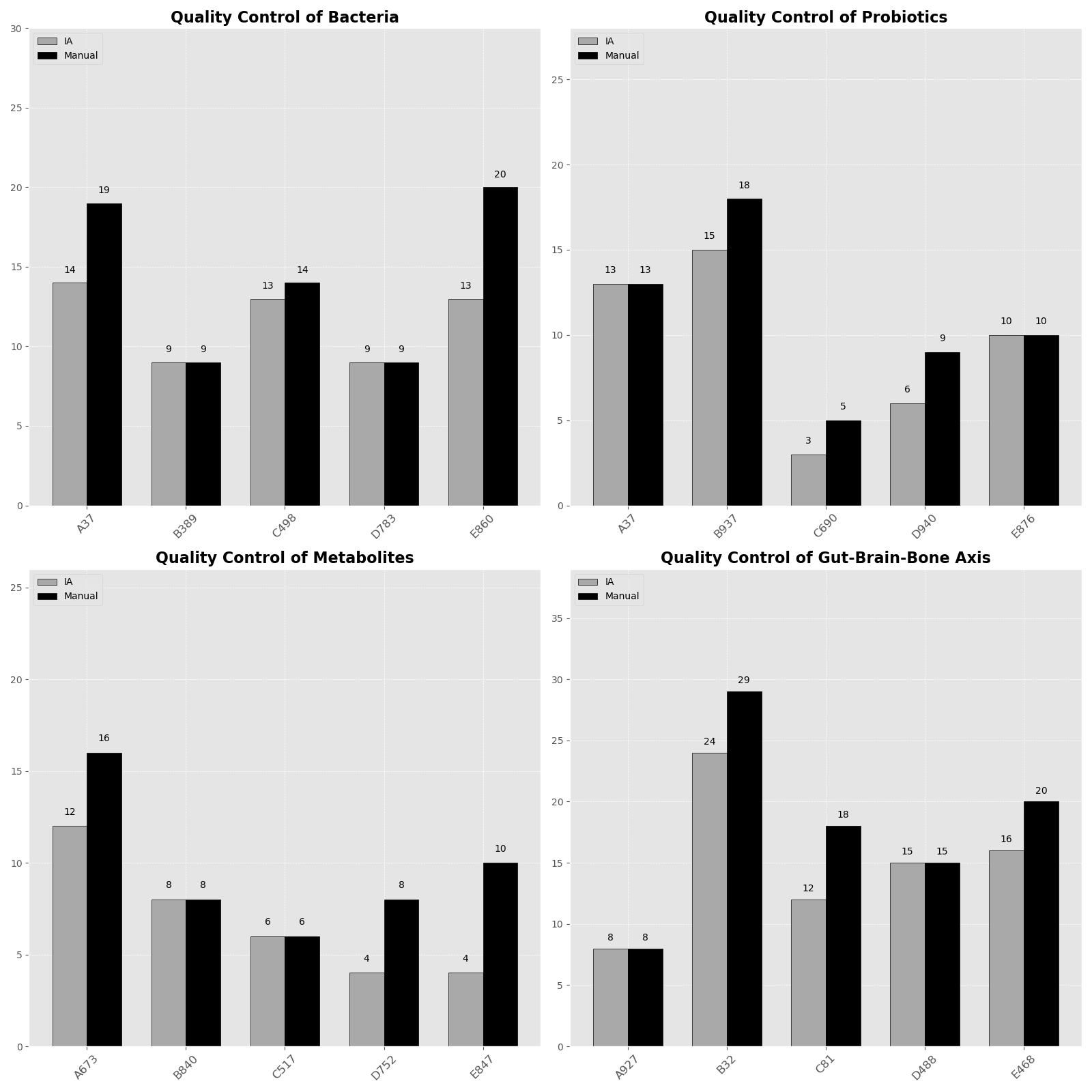


**Figure S1.** *Comparison of results obtained by AI and manual review*


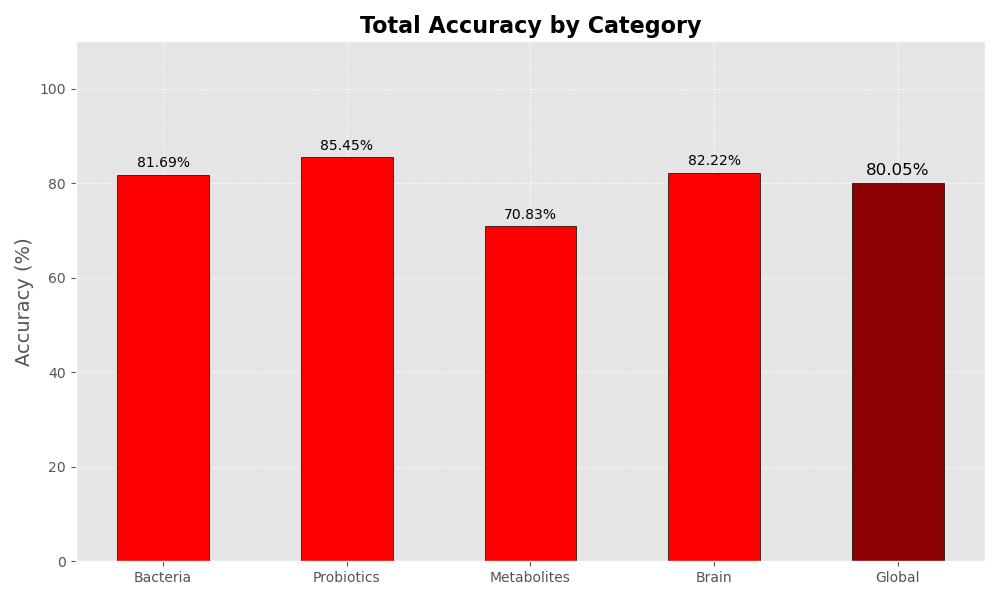


**Figure S2.** Total accuracy percentage in each category of tables.

##
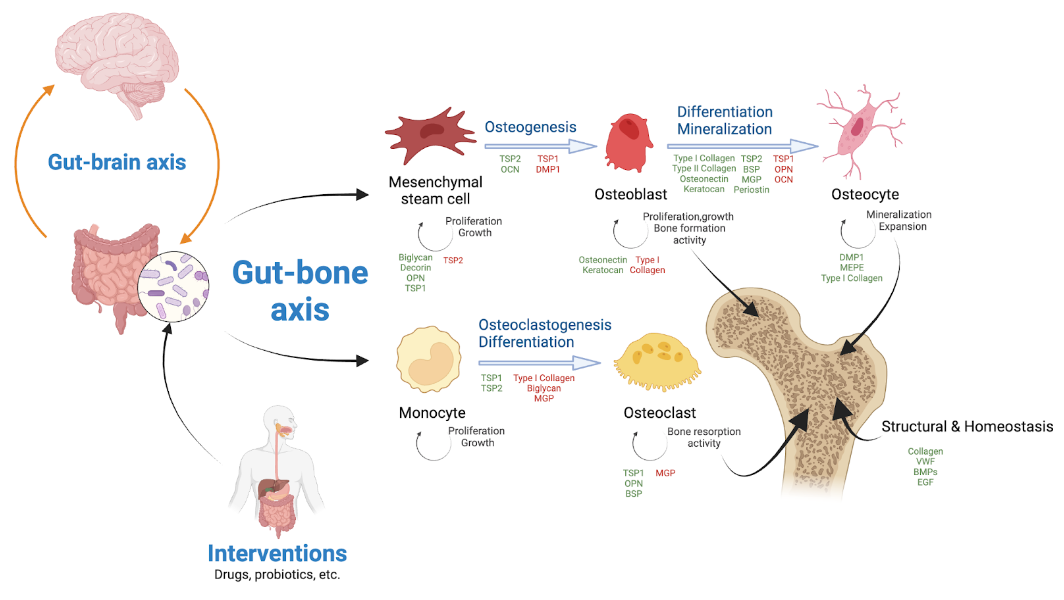


**Figure S3.**  Illustration of the gut-bone axis and its interaction with the gut-brain axis in bone homeostasis (modified with permission [[1]](https://sciwheel.com/work/citation?ids=15360274&pre=&suf=&sa=0))


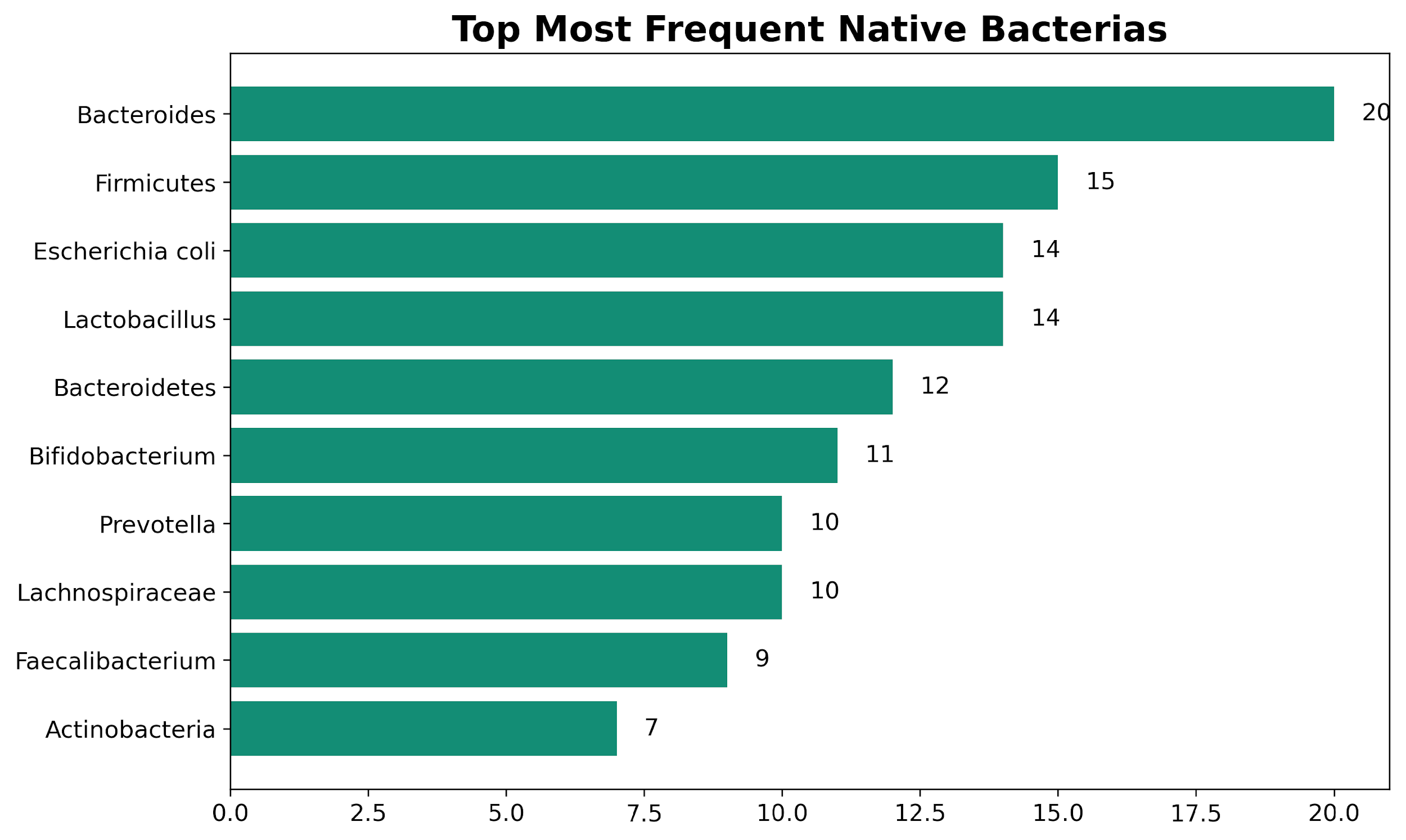


**Figure S4.** Top most frequent native bacteria in the human gut microbiota.


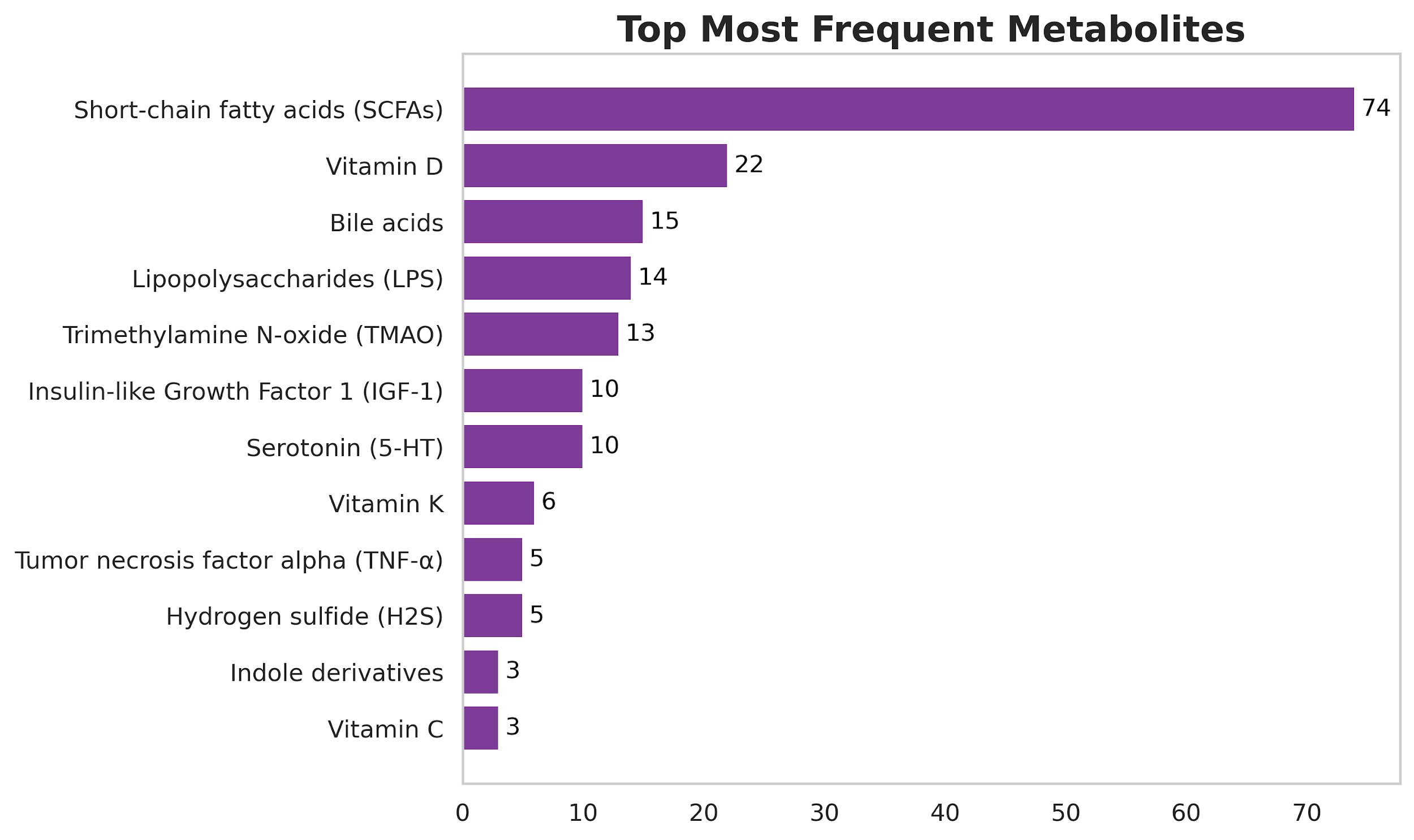


**Figure S5.** Top most frequent metabolites.


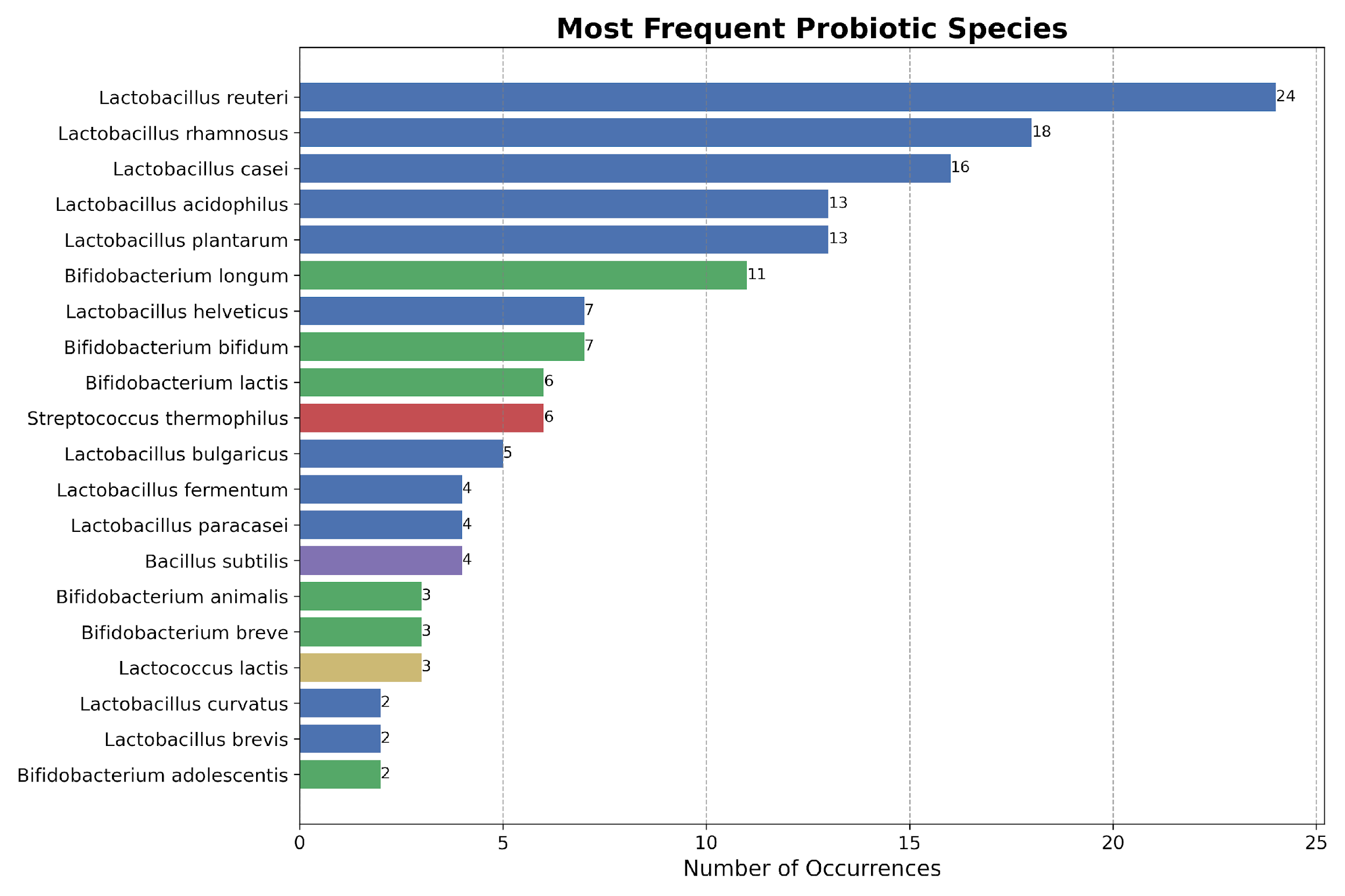


**Figure S6.** Most frequent probiotics (Genus and Species). This figure shows the most frequently identified probiotic species. Lactobacillus (blue), Bifidobacterium (green), Streptococcus (red), and Bacillus (purple).


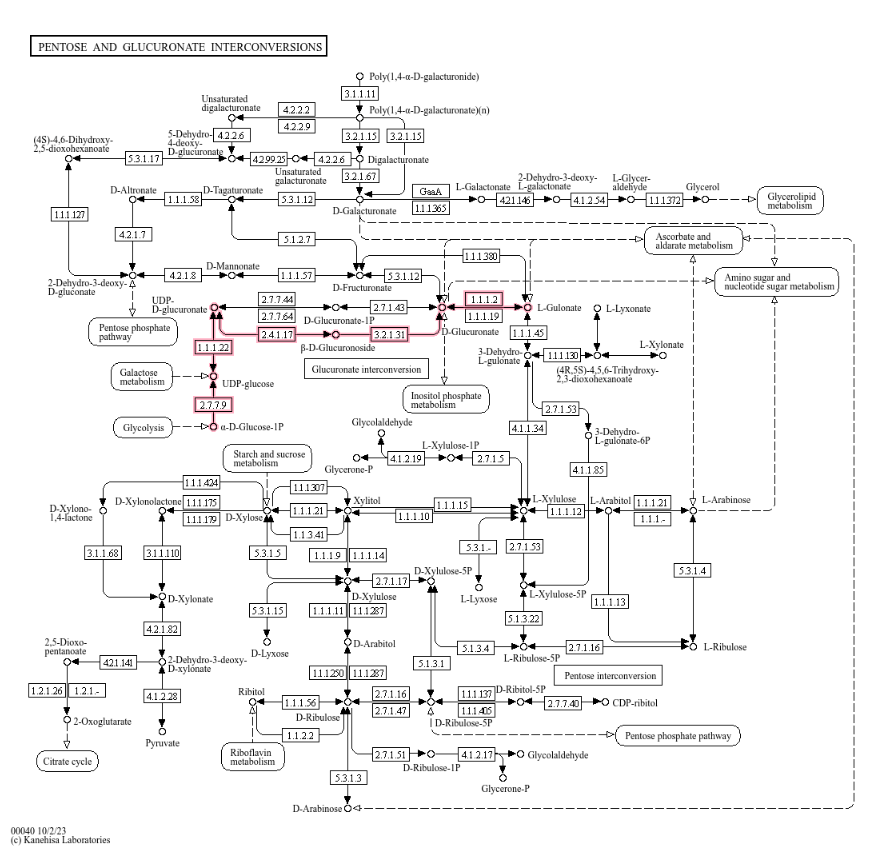


**Figure S7.** LGG metabolic pathways image. Pentose and glucuronate interconversions pathway (map00040).


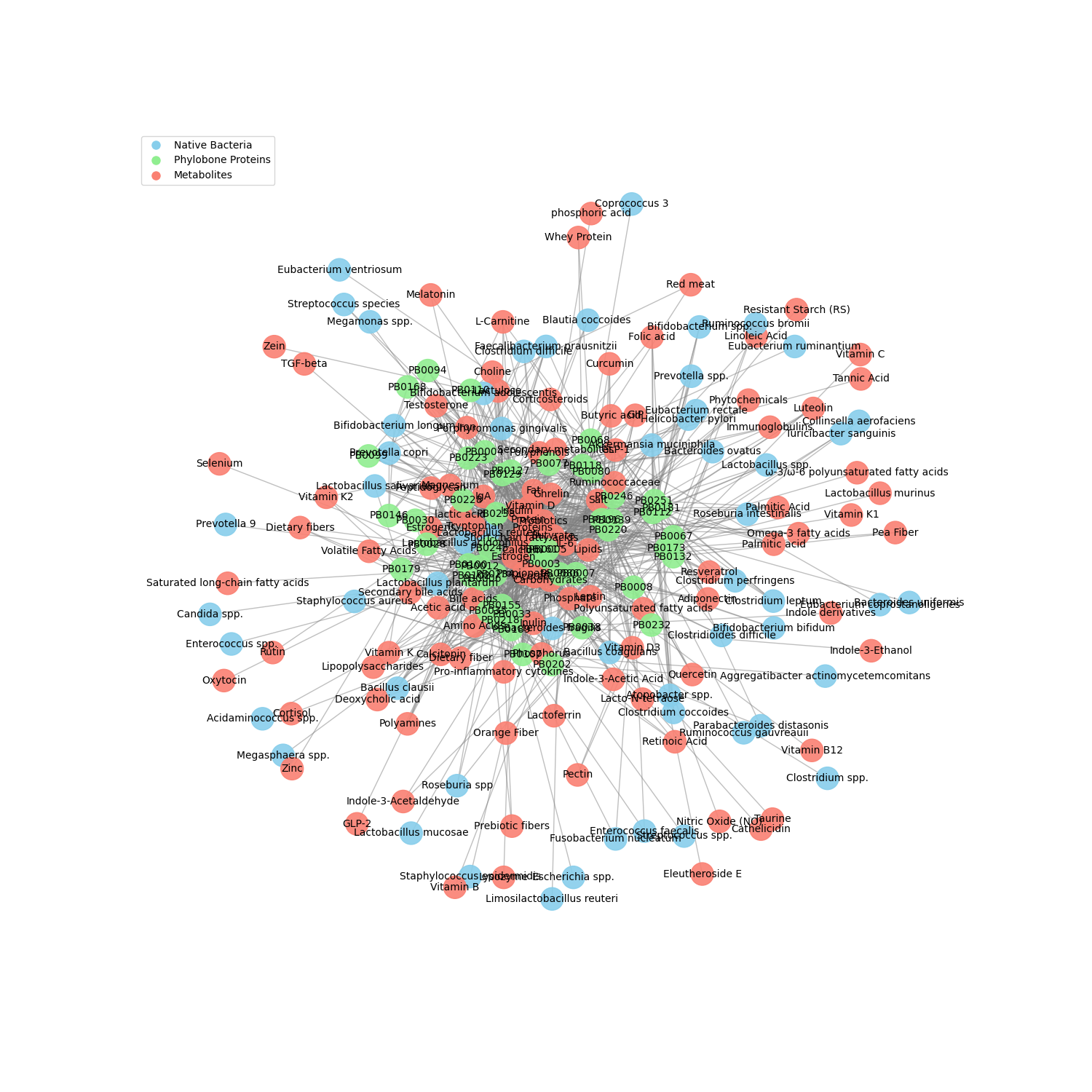


**Figure S8.** Phylobone network illustrates the relationships between gut microbiota (blue nodes), metabolites (red nodes), and ECM proteins(green nodes).

##

## Supplementary Tables

**Table S1.** Summary of articles analyzed. For native bacteria and metabolic pathways, articles with more than 4 keyword matches were prioritized.

| **Category** | **Total Articles** | **Keyword Matches** | **Articles Obtained** |
| --- | --- | --- | --- |
| Native bacteria | 304 | 206 ($x>4$) | 159 |
| Probiotic strains | 86 | 86 | 77 |
| Metabolic pathways | 247 | 217 ($x>4$) | 194 |
| Gut-brain-bone interactions | 35 | 35 | 23 |

**Table S2.** Frequency of relevant proteins according to the Phylobone database, with corresponding PBID

| **Protein** | **PBID** | **Frequency** |
| --- | --- | --- |
| Cathepsin K | PB0100 | 22 |
| Osteopontin | PB0102 | 18 |
| Fibroblast growth factor 23 | PB0181 | 5 |
| Plasminogen | PB0238 | 5 |
| Matrix GLA protein | PB0004 | 4 |
| Matrix metalloproteinase-9 | PB0107 | 4 |
| Apolipoprotein E | PB0179 | 3 |
| Glyceraldehyde-3-phosphate dehydrogenase | PB0011 | 2 |

## Supplementary Datasets

**Dataset 1 - Microbiota**: This dataset focuses on the relationship between different microbiota species and bone health. It provides information about their origin and how they affect bone metabolism, including positive and negative impacts. Also discuss the role of the microbiota in influencing bone density, bone mineralization, and bone formation or resorption through different pathways.

**Dataset 2 - Metabolites**: This dataset provides details on various metabolites derived from the microbiota and their impact on bone health. It describes the origins of these metabolites, their metabolic pathways, and their effects on bone formation, mineral density, and other related processes. The dataset highlights both beneficial and negative impacts, illustrating how these metabolites interact with bone metabolism.

**Dataset 3 - Probiotics**: This dataset highlights various probiotic strains, their origins, and their effects on bone health. It outlines the specific functions of each probiotic, such as enhancing bone mineral density, promoting mineral absorption, or reducing inflammation. The dataset also notes the pathways these probiotics influence, showing their potential to improve bone health by modulating immune responses, inflammation, and gut health.

**Dataset 4 - Bone ECM Proteins**: This Dataset 4 acts as a relational table, facilitating the connection of information on bacteria, ECM proteins from the Phylobone Database, metabolites, and scientific references, offering a valuable resource for analyzing microbial interactions and their impact on bone health.

**Supplementary references**

[1. Fontcuberta-Rigo, M.; Nakamura, M.; Puigbò, P. Phylobone: a comprehensive database of bone extracellular matrix proteins in human and model organisms. *Bone Res.* **2023**, *11*, 44, doi:10.1038/s41413-023-00281-w.](https://sciwheel.com/work/bibliography/15360274)
